# Collodion in Chilblains

**Published:** 1851-03

**Authors:** John Evans

**Affiliations:** Prof. of Obstetrics, &c., in Rush Medical College


### ARTICLE VII

*Collodion in Chilblains.* By Jno. Evans M. D., Prof. of Obstetrics, &c., in Rush Medical College.

Chilblains result from the excess of reaction, following exposure to cold, and in the early stage may be regarded as simply congestions of the parts. The hyperemia affecting the skin, brings the blood so closely in contact with the air, that oxygenization in an unnatural degree goes on, feeding the flame, and producing the intolerable itching and burning sensations characteristic of this affection, and the troublesome inflammation that generally follows.

To diminish the congestion and protect the surface from exposure to the atmosphere, seeming to be the prominent points to be attended to in the treatment in the early stage, I have resorted to the application of the etherial solution of gun cotton in a number of cases; which by its contractility and compression upon the part filled the first; and by its close adaptation to the surface, and the imperviousness to the air of the coating that it made, answered well the other indication.

I have been highly gratified by the results of this practice. The suffering of the patient being promptly relieved and the progress of the diseased action speedily arrested.

Feb. 3, 1851.

